# Machine learning assisted masking of parasitic signals in Bragg coherent diffraction imaging

**DOI:** 10.1107/S160057752501152X

**Published:** 2026-01-30

**Authors:** Ewen Bellec, Steven J. Leake, Mor Levi, Eugen Rabkin, Tobias U. Schülli, Marie-Ingrid Richard

**Affiliations:** a Université Grenoble Alpes CEA Grenoble, IRIG, MEM, NRS 17 Rue des Martyrs F-38000Grenoble France; b ESRF – The European Synchrotron, 38000Grenoble, France; c Department of Materials Science and Engineering Technion-Israel Institute of Technology 3200003Haifa Israel; RIKEN SPring-8 Center, Japan

**Keywords:** Bragg coherent diffraction imaging, machine learning, clustering, BCDI

## Abstract

Bragg coherent diffraction imaging (BCDI) is a lens-less technique capable of imaging the strain in a particle in the size range from 20 nm up to several micrometres. This indirect measurement technique, used in X-ray synchrotrons or free-electron lasers all over the world, requires an inversion step using iterative algorithms in order to recover the real-space complex object encoding the particle shape and deformation field. However, artefacts such as scattering peaks called ‘aliens’ from nearby particles can affect the accuracy of the final reconstruction and require meticulous and time-consuming manual masking of the raw data. This becomes problematic for BCDI reconstructions during an experiment and/or for large volumes of data. Here, we explore the potential of machine learning, and specifically clustering techniques, to speed up this procedure while keeping the maximum spatial resolution of the object reconstruction. We also provide a user-friendly Python *Jupyter* notebook program available on Github.

## Abstract 2

Bragg coherent diffraction imaging measurement sometimes requires manual and time-consuming cleaning of parasitic signals termed ‘aliens’ from nearby particles that can affect the phase retrieval reconstruction. Here, we propose using a clustering technique to speed up this process while keeping the resolution of the reconstructed object high. A user-friendly Python *Jupyter* notebook program is available on Github.

## 1. Introduction

Coherent diffraction imaging (CDI) first emerged in the 2000s (Miao *et al.*, 2000 ▸; Miao *et al.*, 2001 ▸). CDI is a lens-less technique that uses a highly coherent beam to illuminate a sample and generate a diffraction pattern. The far-field scattered beam corresponds to the Fourier transform of the measured object. However, as standard X-ray detectors measure only the diffracted intensity, the phase information is lost. Therefore, iterative feedback algorithms like Error-Reduction and Hybrid-Input-Output (Gerchberg & Saxton, 1972 ▸; Fienup, 1978 ▸; Fienup, 1982 ▸) are used to reconstruct the corresponding real-space object. The main advantage of CDI lies in its resolution, limited only by the X-ray wavelength, the photon dose and, in practice, the extent of scattering in reciprocal space.

In the case of CDI in the Bragg condition (BCDI), the phase of the reconstructed object corresponds to the atomic displacement field projected onto the Bragg wavevector direction, allowing for three-dimensional (3D) strain field imaging on a nanometric scale (Robinson & Harder, 2009 ▸). This powerful technique has been widely used for *in situ* and *operando* imaging of 3D nanomaterials such as during catalytic gas reaction (Ulvestad *et al.*, 2016 ▸; Kim *et al.*, 2018 ▸; Dupraz *et al.*, 2022 ▸; Abuin *et al.*, 2019 ▸; Dupraz *et al.*, 2022 ▸), during battery charging (Singer *et al.*, 2018 ▸) or under electro­chemical conditions (Atlan *et al.*, 2023 ▸).

CDI and its Bragg variant rely heavily on iterative phase retrieval algorithms that are sensitive to the data quality and to the presence of experimental artefacts such as detector gaps or detector noise. In particular, in BCDI there are several perspectives for improving the quality of the reconstruction, as demonstrated by Carnis *et al.* (2019 ▸). Improvements can be made owing to (i) preprocessing steps such as centring, flatfield correction or masking the detector gap and (ii) post-processing steps including data orthogonalization [see for instance Newton *et al.* (2010 ▸, 2012 ▸), Maddali *et al.* (2020 ▸) and Simonne *et al.* (2022 ▸)].

A typical measurement artefact in BCDI is the presence of parasitic signals (termed aliens) in the diffraction pattern, produced by neighbouring particles in the beam tails or even cosmic rays. These can cause large oscillations in the strain field of the reconstructed object and can even prevent the reconstruction from converging. These artefacts are often removed using handmade masks (Carnis *et al.*, 2019 ▸) but this method is time consuming, which becomes problematic for BCDI reconstructions. Fast and reliable data cleaning becomes a necessity with the advent of fourth-generation synchrotrons and the availability of high-coherence X-ray beams (Björling *et al.*, 2019 ▸; Richard *et al.*, 2022 ▸; Atlan *et al.*, 2023 ▸), giving the possibility of continuous BCDI scans (Li *et al.*, 2020 ▸).

Recently, a machine learning (ML) clustering method was proposed as a solution (Pelzer *et al.*, 2021 ▸). This automated method was shown to be much faster than a handmade mask, but it removes high **q** values having low signal intensity, thus impacting the spatial resolution of the reconstruction. Our method accelerates BCDI data pre-processing, while ensuring minimal loss of spatial resolution in the reconstructed object.

Advanced sample preparation techniques, such as patterning to separate individual crystals, can help avoid diffraction pattern overlap. However, such preparation is not always feasible for ‘real-world’ samples, making a method for alien masking essential. Here, we present a clustering-based method to mask alien artefacts selectively, while preserving the integrity of the remaining diffraction signal. Contrary to the work of Pelzer *et al.* (2021 ▸), our approach focuses solely on eliminating the signal attributed to the aliens without removing the high-**q** signal. This selective masking ensures that any loss of real-space resolution is minimized.

Our method requires minimal user interaction for cluster selection and no fine-tuning. It is very efficient for fast alien masking during an experiment. In this paper we outline the preprocessing steps which lead to the creation of individual cluster masks, describe the interactive user-driven cluster selection process, and finally show the result of this masking on two experimental data reconstructions, the first containing numerous aliens and the second exhibiting a strong diffuse scattering peak. Additionally, we include a benchmark comparative study between our method and the code developed by Pelzer *et al.* (2021 ▸).

## 2. Methods

### 2.1. Pipeline

**Figure 1:**
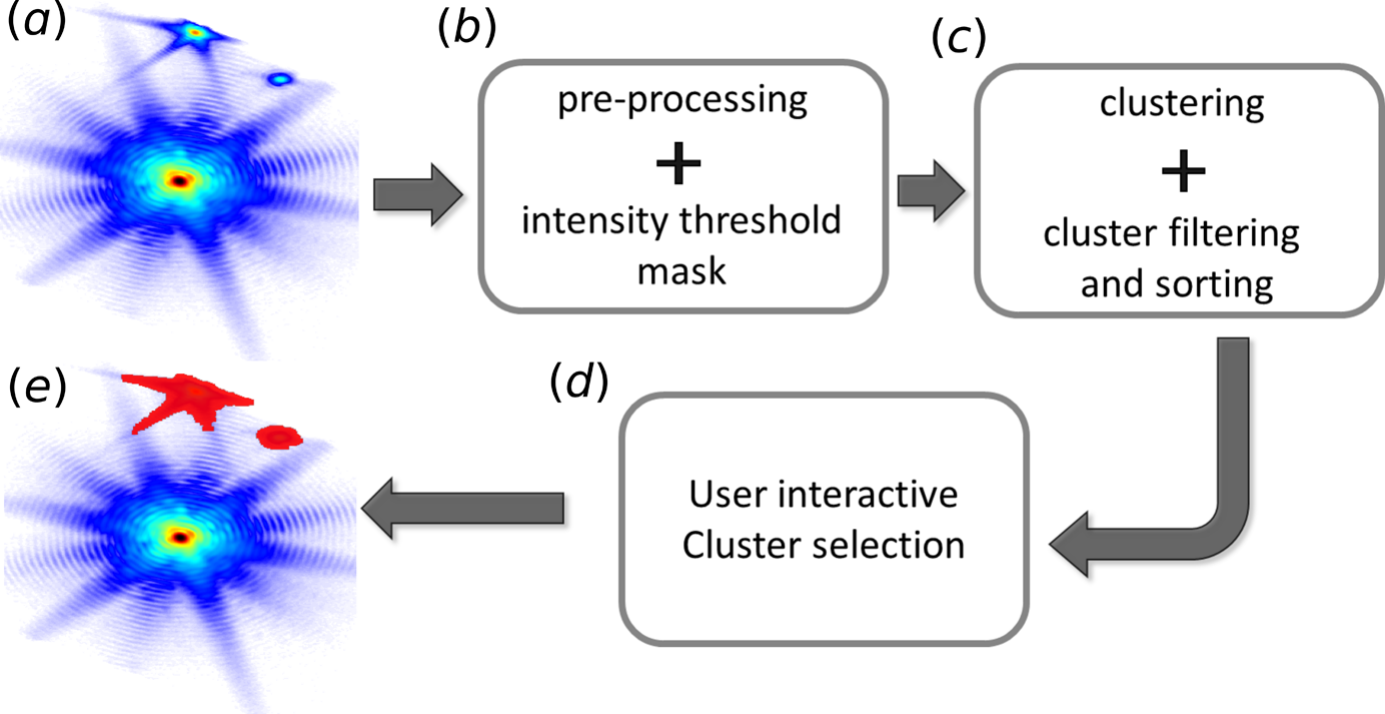
Pipeline for the alien mask clustering. (*a*) Centred 3D BCDI data containing two aliens in the top part of the image. (*b*) Preprocessing including hot + low pixel filtering and log-rescaling, followed by a transformation of the BCDI array into a list of pixel positions using an intensity threshold mask. (*c*) DBSCAN clustering followed by filtering and sorting steps. (*d*) User selection of the alien clusters. (*e*) Alien mask output.

The alien masking pipeline is illustrated in Fig. 1 ▸. A typical centred 3D BCDI array (**I**) containing aliens is used as input [Fig. 1 ▸(*a*)]. This array is then preprocessed to return a log-scale data array, from which an intensity threshold mask (**M**_th_) is made. **M**_th_ is then transformed into a list of pixel positions and clustered with the Density-Based Spatial Clustering of Applications with Noise (DBSCAN) algorithm (Ester *et al.*, 1996 ▸) of the Python module *scikit-learn* (Pedregosa *et al.*, 2011 ▸). The clusters are then filtered to remove the central Bragg peak and isolated noise pixels, and finally sorted to have the alien clusters placed among the first positions with high probability. The user then hand-picks the relevant alien clusters using a Python *Jupyter* notebook widget interface. Finally, these selected clusters are combined in order to create the complete alien mask.

### 2.2. Data preprocessing

**Figure 2:**
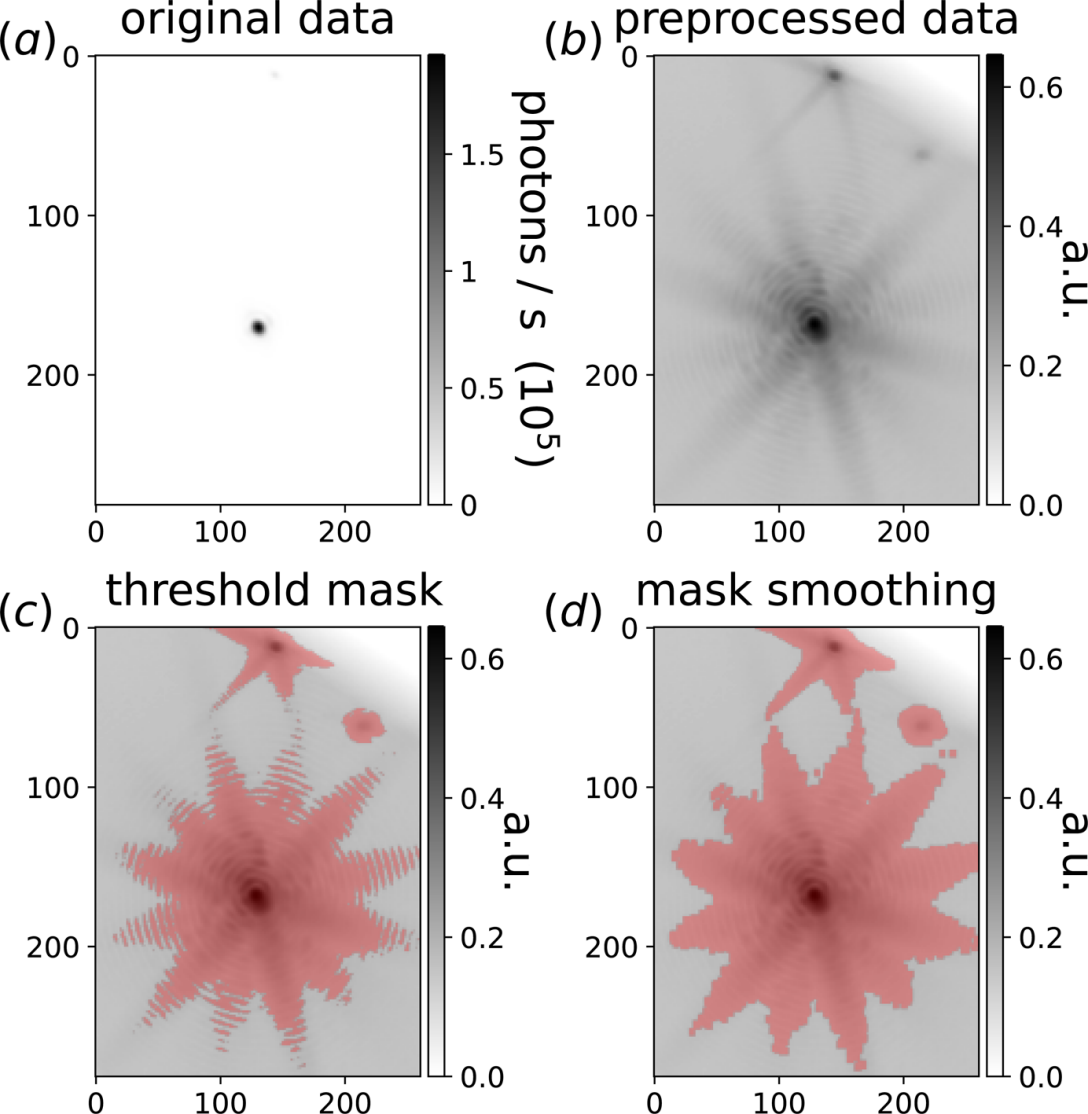
Step-by-step preprocessing. (*a*) Linear scale projection of the 3D BCDI data along the first dimension. (*b*) Hot + low pixel filtering followed by a custom log-rescaling. (*c*) Intensity threshold mask **M**_th_ in red. (*d*) Mask smoothing using a maximum filter in order to merge the Bragg peak fringes.

The preprocessing steps are detailed in Fig. 2 ▸. The 3D BCDI data array (**I**) projection is shown on a linear scale in Fig. 2 ▸(*a*). It contains a very intense central peak. A hot pixel filter, *scipy.ndimage.median_filter*, is applied to the array in order to remove all problematic detector pixels. A low-intensity filter is also applied to remove all pixels with a photon count <1, thus avoiding large negative values during the following log-rescaling step. Finally, our custom rescaling is used in order to return bounded data on a logarithmic scale between 0 and 1 (**I**_log_), as shown in equation (1): 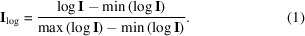
The result of this preprocessing is shown in Fig. 2 ▸(*b*), where the two aliens are now clearly visible at the top of the image. A first mask (**M**_th_) is created, defined by
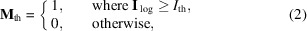
where *I*_th_ is the user-input intensity threshold ranging from 0 (all pixels in the mask) to 1 (no pixels in the mask). **M**_th_ is shown in red in Fig. 2 ▸(*c*) for *I*_th_ = 0.2. In order to separate the alien signal from the central peak into different clusters, the mask intensity threshold should be large enough such that **M**_th_ does not cover the overlapping region, as shown in Fig. 2 ▸(*c*). In practice, no fine tuning is needed and, for typical BCDI data, this threshold is in the range between 0.2 to 0.4 depending on the signal-to-noise ratio.

A problem arises from the fringes of the Bragg peak at high **q** positions, where **q** is the scattering vector. One can observe in Fig. 2 ▸(*c*) gaps between fringes far from the central peak. As shown in Fig. S1 in the supporting information, these fringes are clustered out of the central peak, making the number of clusters very large. To avoid this issue, we use a maximum filter (*scipy.ndimage.maximum_filter*) to smooth the mask, resulting in the merging of the fringes as shown in Fig. 2 ▸(*d*) and in Fig. S2. This results in a drastic decrease in the number of clusters (from 186 to 8 clusters), as shown in Fig. S3. In practice, the maximum filter kernel size is chosen to be between 2 and 5, depending on the number of pixels per fringe. Finally, the intensity threshold mask is transformed into a list (**R**) containing all pixel positions for which **M**_th_ = 1.

### 2.3. Clustering

**Figure 3:**
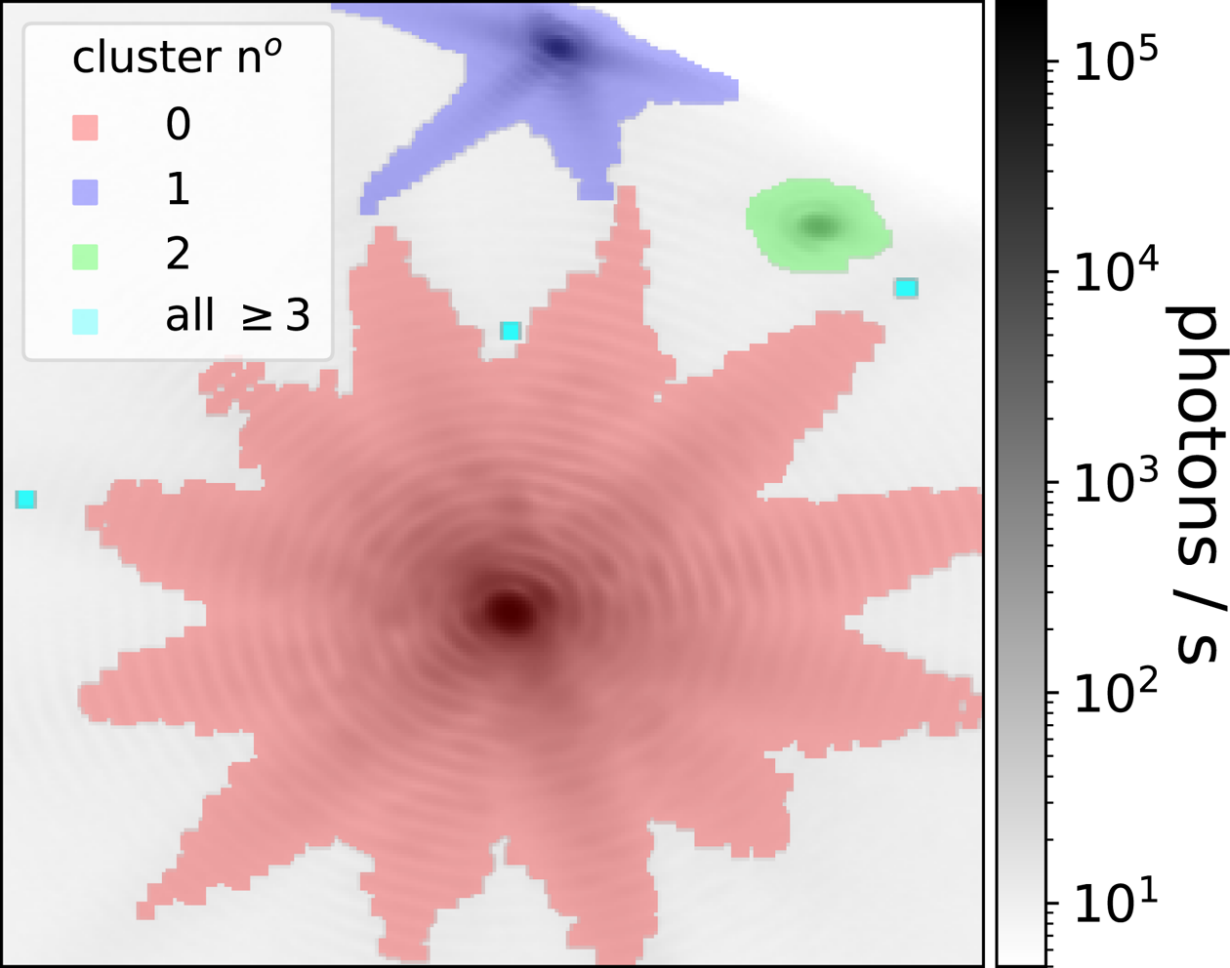
Result of DBSCAN clustering after cluster sorting of BCDI data with two aliens in the top of the image. Both aliens (blue and green clusters) are clustered out from the central Bragg (red cluster). Some unwanted small clusters are also found and shown in light blue, corresponding to Bragg peak fringes and some noisy high-intensity pixels.

DBSCAN is a well known unsupervised clustering technique (Ester *et al.*, 1996 ▸; Schubert *et al.*, 2017 ▸). This density-based algorithm finds points that are closely packed together in a dataset and groups them together. We selected this algorithm for its capability of identifying arbitrarily shaped clusters, since in the case of BCDI data the central peak and aliens can have varying shapes depending on the crystal morphology and its internal strain (Dupraz, 2015 ▸). The result of this clustering on the pixel position array **R** is shown in Fig. 3 ▸. The central Bragg peak cluster is shown in red and the two alien clusters in blue and green. The remaining unwanted clusters are all shown with the same colour in light blue. Although DBSCAN has two free parameters, the standard values *eps* = 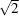 and *min_samples* = 8 are fixed to avoid the need for additional user inputs. Those values were used for all alien masking shown here and in the supporting information.

The clusters are then filtered. First, since BCDI data should in principle be centred, our method removes the cluster containing the central pixel (cluster 0 in red in Fig. 3 ▸), as this is the main Bragg peak and not an alien. Furthermore, we remove noisy isolated pixels that are not part of any clusters. They are automatically found by the *sklearn.cluster.DBSCAN* function. Finally, before the user selects clusters, we sort the clusters in order to have the alien clusters in first positions with high probability. For this, several methods are available, namely ‘size’ to order clusters by their number of pixels, ‘max’ to place the clusters which contain the largest pixel intensity first, and finally ‘asym’ that classifies clusters by their average asymmetry factor as defined by Pelzer *et al.* (2021 ▸) and shown in Fig. S4. In practice, sorting clusters by size is often the best approach.

Despite these preprocessing, filtering and sorting steps, finding the number of aliens and distinguishing between an alien cluster and a central peak fringe in a fully automatic way is too complex and unreliable. In order to make a fast and fully reliable alien masking procedure, an interactive user alien cluster selection is available using *ipywidgets* (https://github.com/jupyter-widgets/ipywidgets) as shown in Fig. S5. For each cluster, a figure containing the BCDI data projection along each of the three axes and the corresponding cluster mask are shown with a checkbox widget associated with each figure. Finally, the alien mask is created by combining the selected clusters.

Our method avoids, as much as possible, any need for user fine tuning of the intensity threshold mask and clustering parameters that would slow down the masking procedure. The preprocessing is general enough to be easily used on BCDI data having very different signal-to-noise ratios, peak shapes, numbers of aliens and background noise, as shown in Figs. S6 and S7.

## 3. Results and discussion

### 3.1. Masking of BCDI data with numerous aliens

The algorithm described above was applied to the 3D diffraction pattern of a 500 nm diameter platinum crystal. The Pt crystals were prepared by the solid-state dewetting of a 30 nm thin Pt film for 24 h at 1100°C in air. The Pt film was deposited on α-Al_2_O_3_ (sapphire) with an electron beam evaporator. The Pt nanocrystals have their [111] direction normal to the (0001) sapphire substrate. A standard photolithography method was employed to prepare a patterned layer of photoresist on the sapphire prior to the electron beam evaporation of Pt. The lithographic processing route ensured that a number of dewetted Pt particles are well separated from their neighbours and that only one crystallite is irradiated by the incoming X-ray beam (Fig. S8). The measured particle was separated by 25 µm from the other crystals.

**Figure 4:**
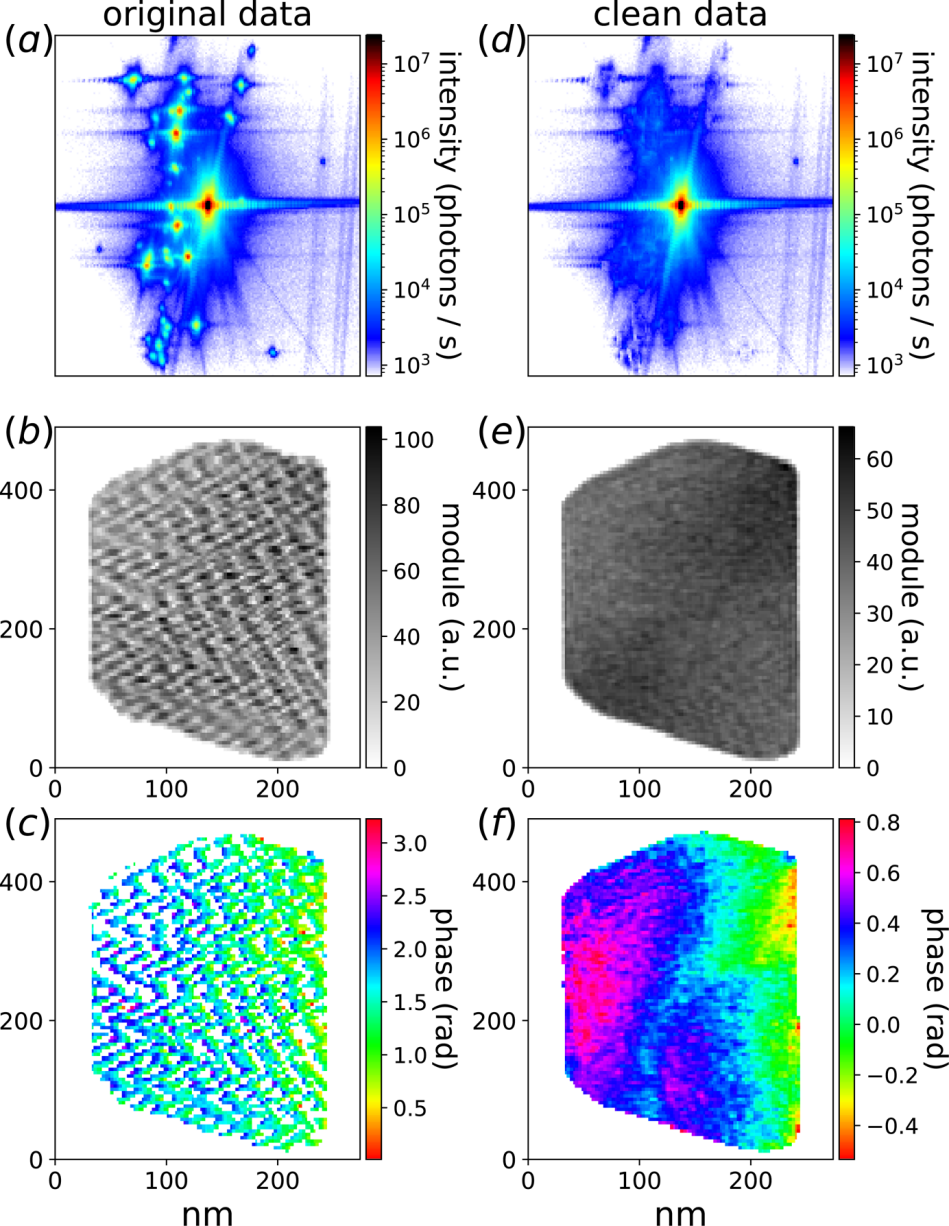
Phasing results of BCDI data with and without aliens. (*a*) Diffraction data containing numerous aliens. (*b*, *c*) Reconstructed object module and phase with strong oscillation artefacts. (*d*) BCDI data after alien masking using our clustering method. (*e*, *f*) Corresponding object reconstruction where most of the oscillations have disappeared.

The experiment was performed on the ID01 beamline at the fourth-generation Extremely Brilliant Source at the European synchrotron (ESRF-EBS, Grenoble, France) using a CITIUS charge-integrating detector (Grimes *et al.*, 2023 ▸). The coherent X-ray beam was focused down to 800 nm using compound refractive lenses at a beam energy of 20 keV. Despite careful sample preparation, the focused beam tails still illuminate other nearby particles (see Section S15 of the supporting information), leading to the numerous aliens observed in Fig. 4 ▸(*a*).

In some cases, with either intense and/or numerous aliens, BCDI reconstruction becomes impossible. For the data shown in Fig. 4 ▸(*a*), reconstruction of the object is still possible using iterative phasing algorithms (Favre-Nicolin *et al.*, 2020 ▸), but the presence of many aliens leads to strong oscillatory artefacts in the modulus and phase of the reconstructed particle [Figs. 4 ▸(*b*) and 4 ▸(*c*)]. Moreover, the surface of the particle becomes less defined, as illustrated in Figs. S9(*b*) and S9(*d*). This highlights why the presence of signals from aliens poses challenges when tracking particle evolution during *in situ* electrochemistry (Atlan *et al.*, 2023 ▸) or gas-phase experiments (Ulvestad *et al.*, 2016 ▸; Kim *et al.*, 2018 ▸; Abuin *et al.*, 2019 ▸; Kawaguchi *et al.*, 2019 ▸; Dupraz *et al.*, 2022 ▸), particularly where chemical reactions occur near the surface. Finally, in cases with few and/or less intense aliens (Fig. S10), these oscillations could in principle be removed with an apodization (Carnis *et al.*, 2019 ▸) but this leads to a loss of spatial resolution.

Our cluster alien masking method was used to locate the aliens in a BCDI data array and replace them by zeros as shown in Fig. 4 ▸(*d*). One can still observe part of the alien signals due to the limitation of our intensity threshold masking shown in Fig. 2 ▸. Nevertheless, most of the alien intensity is removed by our method as shown in Fig. S11, and the object reconstruction does not show these strong oscillation artefacts anymore [Figs. 4 ▸(*e*) and 4 ▸(*f*)]. The spatial resolution, after alien removal, is calculated with the Fourier correlation shell using two independent reconstructions, which gives a resolution of 5.8 nm as shown in Fig. S13.

We must emphasize that even though a handmade masking can be done with more precision on the alien’s low-intensity regions, our method is much faster, taking only one or two minutes depending on the number of alien clusters, and thus being very convenient for phasing during an experiment or for a large set of data containing aliens.

### 3.2. Masking BCDI data with intense aliens

**Figure 5:**
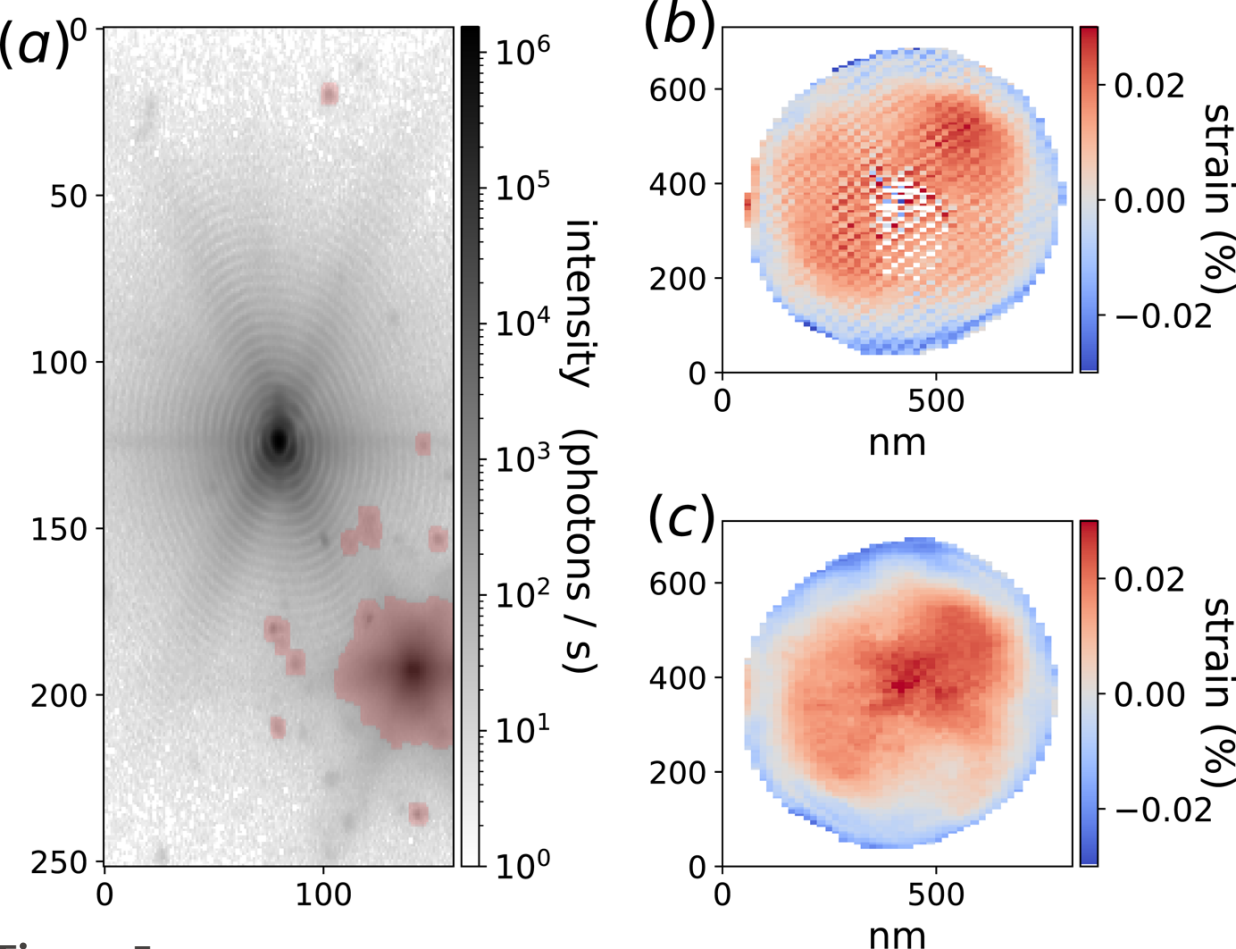
(*a*) Pt 111 Bragg peak projection with an intense diffuse scattering peak in the bottom right of the picture coming from the scattering of neighbouring particles. The alien clustering mask is shown in transparent red. (*b*) Reconstruction of the out-of-plane strain with the alien signal. Large oscillation artefacts are visible, as well as arbitrarily large strain values near the centre. (*c*) Out-of-plane strain reconstruction using the alien mask, in which the artefacts have disappeared.

As a second example, our method was applied to the BCDI data of the 111 Bragg peak of a Pt particle deposited on a sapphire substrate. The particle is slightly misoriented with respect to the substrate and its isolated Bragg peak was positioned at the centre of the array as shown in Fig. 5 ▸(*a*). An intense diffuse scattering peak is visible at the bottom right of the figure due to nearby Pt particles having the same orientation as the substrate. This peak induces large oscillations on the modulus and phase of the reconstructed object (Fig. S9). The out-of-plane strain [Fig. 5 ▸(*b*)] of the particle, being the object phase gradient projection along the Bragg peak wavevector, also contains these artefacts. Furthermore, these oscillations cause problems during the phase unwrapping of the object, leading to artificially large strain values close to the centre of the array. Our ML assisted masking method was used to create the alien mask shown in transparent red in Fig. 5 ▸(*a*). The associated out-of-plane strain recovered from phase retrieval is shown in Fig. 5 ▸(*c*). Despite the fact that our mask does not cover the diffuse alien peak entirely, the large intensity portion is removed and the reconstruction artefacts have disappeared.

### 3.3. Benchmark comparative study

In Section S12 of the supporting information, we provide a comparative study between our method and the *auto_alien1* code developed by Pelzer *et al.* (2021 ▸). As shown in Fig. S14, the default set of parameters of *auto_alien1* does not allow it to catch all parasitic alien signals in difficult data with noisy background (fluorescence scattering) or containing a large number of aliens. Although the code does not require any user interaction, it does sometimes require fine tuning of the input parameters, as shown in Figs. S15 and S16. We have also compared *auto_alien1* with our method on a simulated object in Figs. S17, S18 and S19. We show that our method is able to remove the alien signals on the BCDI data reliably, while minimizing any degradation of the spatial resolution in the reconstructed object.

## 4. Conclusion

Here, we have demonstrated the application of machine learning assisted masking of parasitic signals in Bragg coherent diffraction imaging while minimizing any loss of spatial resolution in the reconstructed particle. Our method avoids fine-tuning operations and we provide a user-friendly Python *Jupyter* notebook code available on Github (see *Data availability* section).

We have shown that this technique can be used on very different types of BCDI data, including low signal-to-noise measurements and asymmetric Bragg peaks. We have confirmed that our masking method removes alien oscillatory artefacts from the reconstructed object.

This method overcomes meticulous and time-consuming handmade masking of the raw data. With the significant increase in BCDI data production provided by fourth-generation synchrotron light sources, this improvement in efficiency will help BCDI data processing.

## Supplementary Material

Video tutorial. DOI: 10.1107/S160057752501152X/yn5122sup1.mp4

Supporting information Sections S1 to S15, including Figures S1 to S21. DOI: 10.1107/S160057752501152X/yn5122sup2.pdf

## Data Availability

Data are available in the reports by Dassonneville *et al.* (2023 ▸) and Bellec *et al.* (2023 ▸). The Python code, along with a *Jupyter* notebook and a typical example of BCDI data, are available on Github at https://github.com/ewbellec/alienclustering. A video tutorial is provided in the supporting information.
