# Supplementary material for: Machine learning assisted masking of parasitic signals in Bragg coherent diffraction imaging
Source: J Synchrotron Radiat. 2026 Jan 30;33(Pt 2):448–53. doi: 10.1107/S160057752501152X (PMC12948010; doi:10.1107/S160057752501152X)
Supplement: Supplementary file 2 [file s-33-00448-sup2.pdf]

JOURNAL OF SYNCHROTRON RADIATION

Volume 33 (2026)

Supporting information for article:

Machine learning assisted masking of parasitic signals in Bragg coherent diffraction imaging

Ewen Bellec, Steven J. Leake, Mor Levi, Eugen Rabkin, Tobias U. Schülli and Marie-Ingrid Richard

Supplementary Section 1: Fringes clustering problem

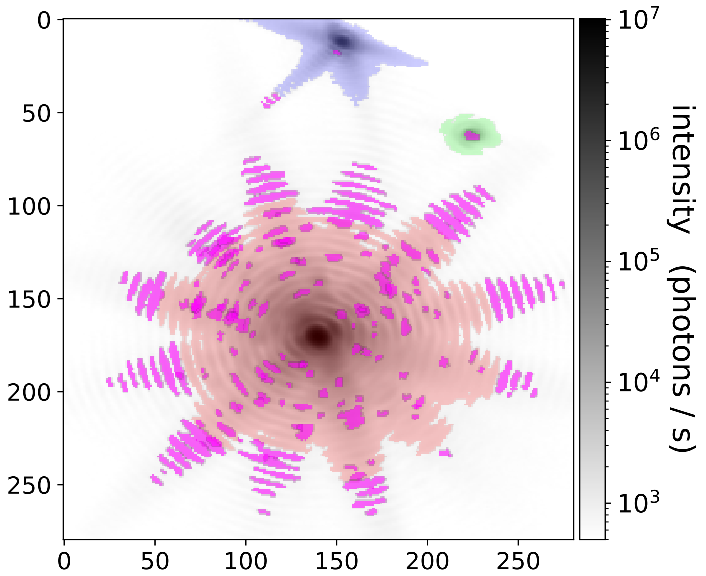

Supplementary Figure 1: DBSCAN clustering of BCDI data containing aliens. The three main clusters, shown in light red, blue and green correspond to the central Bragg peak and the two aliens, respectively. The other clusters are all shown with the same color (pink) and are mainly due to the fringes of the central peak.

DBSCAN is a density-based clustering technique that can divide dense regions of space separated by lower density regions. This is typically the case for BCDI data involving aliens, where the alien peaks often remain disconnected from the main Bragg peak. Unfortunately, in BCDI data with a high signal-to-noise ratio, the fringes of the main peak are separated by regions of low intensity, causing them to be excluded from the main Bragg peak during clustering as shown in Supplementary Fig. **1**, where many fringes are shown to be in different clusters. This significantly slows down the alien cluster user-selection procedure, as the presence of these fringes greatly increases the number of clusters identified by the DBSCAN algorithm. In principle, using DBSCAN from the *sklearn.cluster* module, one could solve this issue by fine-tuning the clustering parameters *eps* and *min_samples* but this is inefficient in practice for a simple and user-friendly BCDI masking.

Supplementary Section 2: Fringes mask smoothing

A simple solution to the central peak fringes problem is to use a maximum filter (*maximum_filter* function from the *scipy.ndimage* module) in order to smooth the intensity threshold mask $\mathbf{M}_{th}$. For a kernel

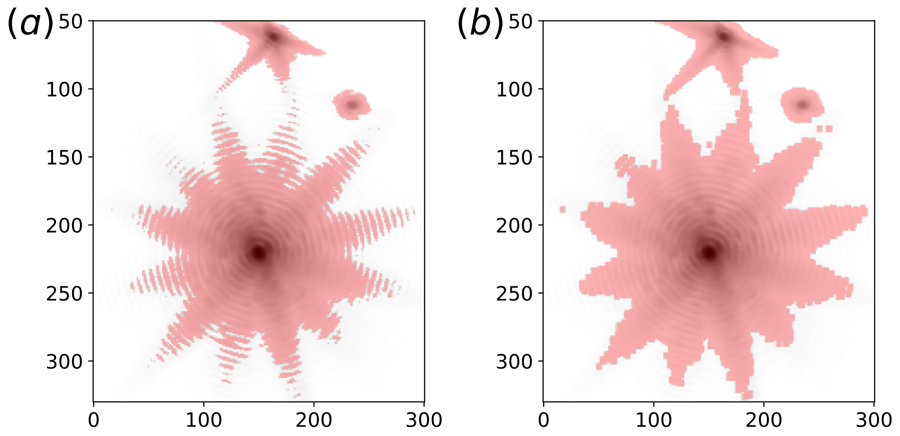

Supplementary Figure 2: (*a*) Intensity threshold mask $\mathbf{M}_{th}$ for pixel selection before clustering. Many fringes are shown to be separated from the central peak. (*b*) Same mask after using a maximum filter with a kernel size of 5.

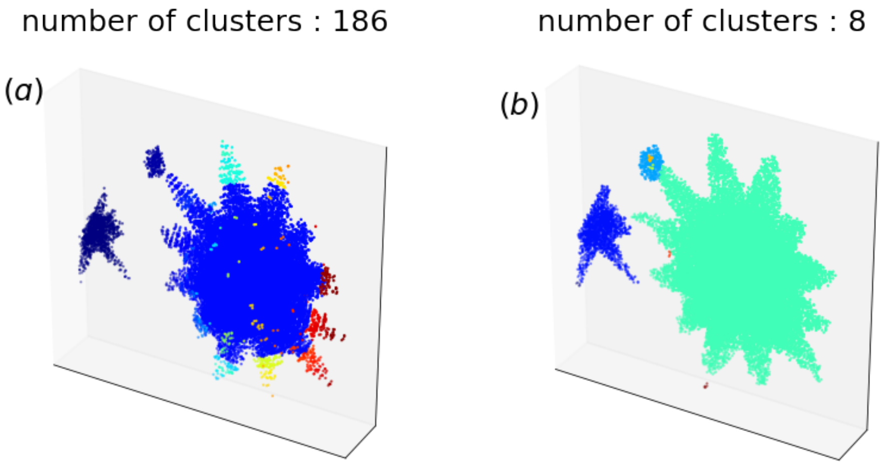

Supplementary Figure 3: (*a*) Clustering using the intensity threshold mask. (*b*) Clustering after smoothing the mask with a maximum filter with kernel size of 5 showing a drastic decrease of the number of found clusters.

size N, this filter forces all pixels in a box of N^d pixels (where d is the array dimension, 3 for our BCDI data) to be equal to the maximum value in this box. In our case, after making $\mathbf{M}_{th}$ shown in red in Supplementary Fig. **2**(*a*), the maximum filter is used with a kernel size σ=5 to smooth out the fringes as shown in Supplementary Fig. **2**(*b*). σ should be roughly the fringe spacing and, in practice, using a value of σ between 2 and 5 is enough and no fine-tuning is needed. σ should not be too large to avoid merging the aliens with the central peak.

This decrease of the number of clusters after this maximum filter mask smoothing is illustrated in Supplementary Fig. **3** with σ=5. In this example, the number of clusters drastically decreases due to

the merging of fringes and only the central Bragg peak remains with two aliens and few noisy pixels.

Supplementary Section 3: Cluster asymmetry

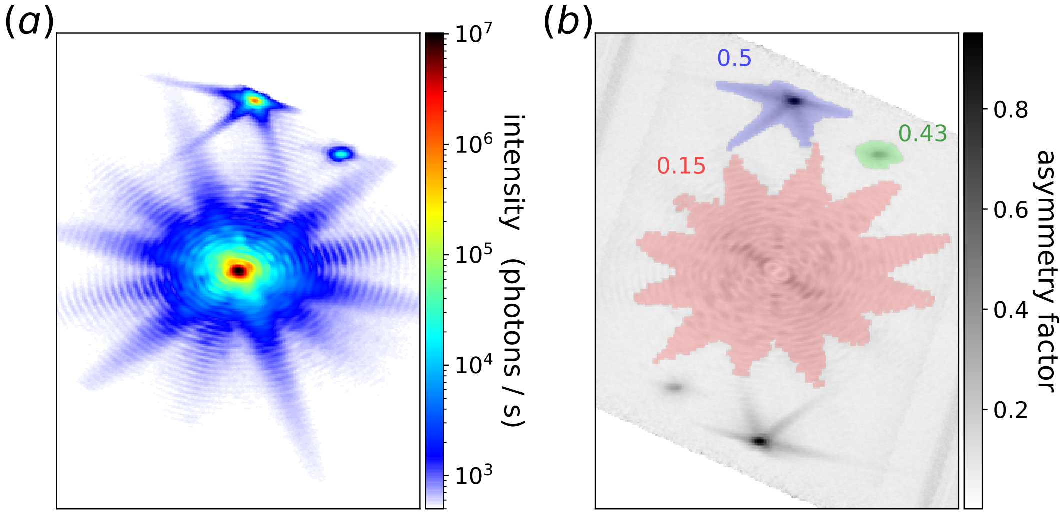

Supplementary Figure 4: (*a*) BCDI data projection along the 2nd dimension. (*b*) Asymmetry matrix in gray scale. The 3 main clusters are superimposed in light red, blue and green along with their average asymmetry factor.

In order to accelerate the interactive cluster selection by the users, a cluster sorting process is applied. Several sorting algorithms are proposed, including sorting by cluster size, by the maximum pixel value within each cluster, and by the average asymmetry factor of the clusters. Given a BCDI array $\mathbf{I}$ shown in Supplementary Fig. **4**(*a*) and its corresponding inverted version $\mathbf{I}_{inv}$ (in python, $\mathbf{I}_{inv}$ = *numpy.flip*($\mathbf{I}$, *axis=(0,1,2)*)), the asymmetry matrix is defined as:

$$\mathbf{A} = 2\frac{\mathbf{I} - \mathbf{I}_{inv}}{\mathbf{I} + \mathbf{I}_{inv}}. \quad (1)$$

$\mathbf{A}$ is shown in Supplementary Fig. **4**(*b*) in gray scale. The alien clusters in light blue and green, having no corresponding intensity at their inverted positions, have a relatively large asymmetry factor (0.5 and 0.43, respectively). On the other hand, the central peak cluster in red, being mostly symmetrical, has a low asymmetry factor of 0.15.

Supplementary Section 4: Cluster selection

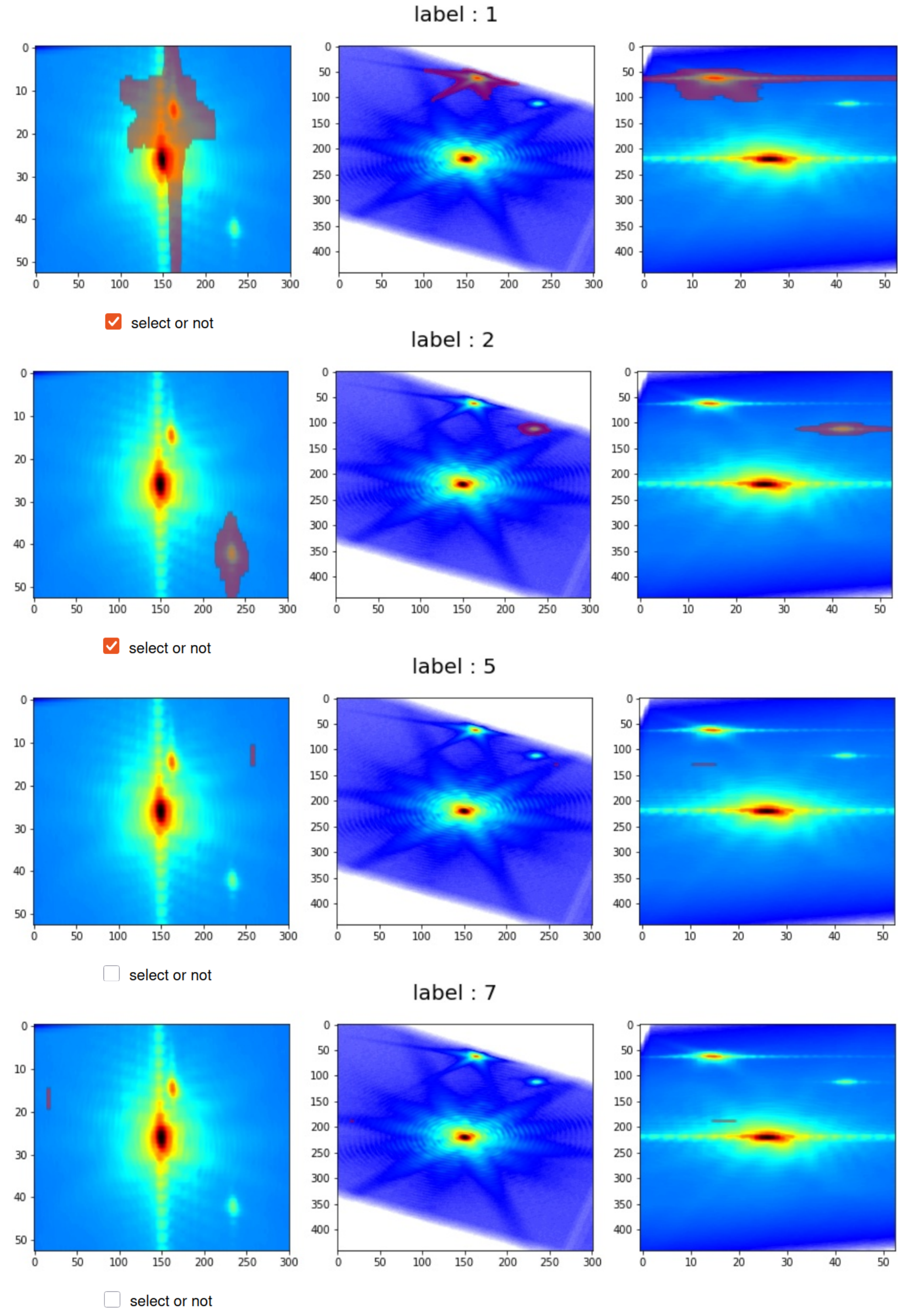

Supplementary Figure 5: User interactive cluster selection shown for a BCDI data with 2 large aliens. The two large alien clusters are among the first positions due to the cluster sorting by cluster size.

Due to the challenges associated with fringes clustering, the cluster selection process cannot be fully automated. Even with the mask smoothing procedure described in Supplementary Section S2, the clustering algorithm tends to identify more clusters than the actual number of aliens, making it challenging to distinguish between alien clusters and clusters associated with the fringes of central

peaks. Therefore, after deciding that a mask containing only the aliens was more important than having a fully automated algorithm, we made a python notebook user interface using the *ipywidgets* module. A typical example is shown in Supplementary Fig. **5**. After intensity threshold masking, mask smoothing and cluster sorting, each cluster is shown individually on 3 sub-figures corresponding to the 3D BCDI data projection along each axis, along with the corresponding cluster projection shown in light red. If a sorting algorithm is used, the alien clusters will be among the first figures with high probability. From these, one can easily distinguish the alien clusters and tick the corresponding checkboxes below each figure. The final alien mask is then made by combining these selected clusters.

Supplementary Section 5: Aliens masking examples

In Supplementary Figure **6**, we show 6 different BCDI data containing aliens with the corresponding masking made using our alien removal python notebook. This shows that our method can be used on very different types of BCDI data having numerous or few aliens, low or high signal-to-noise ratio, with aliens far or close to the central Bragg peak, etc. Furthermore, our clustering-masking is very fast compared to a handmade masking and conserves all high-q low intensity data even far from the central Bragg peak.

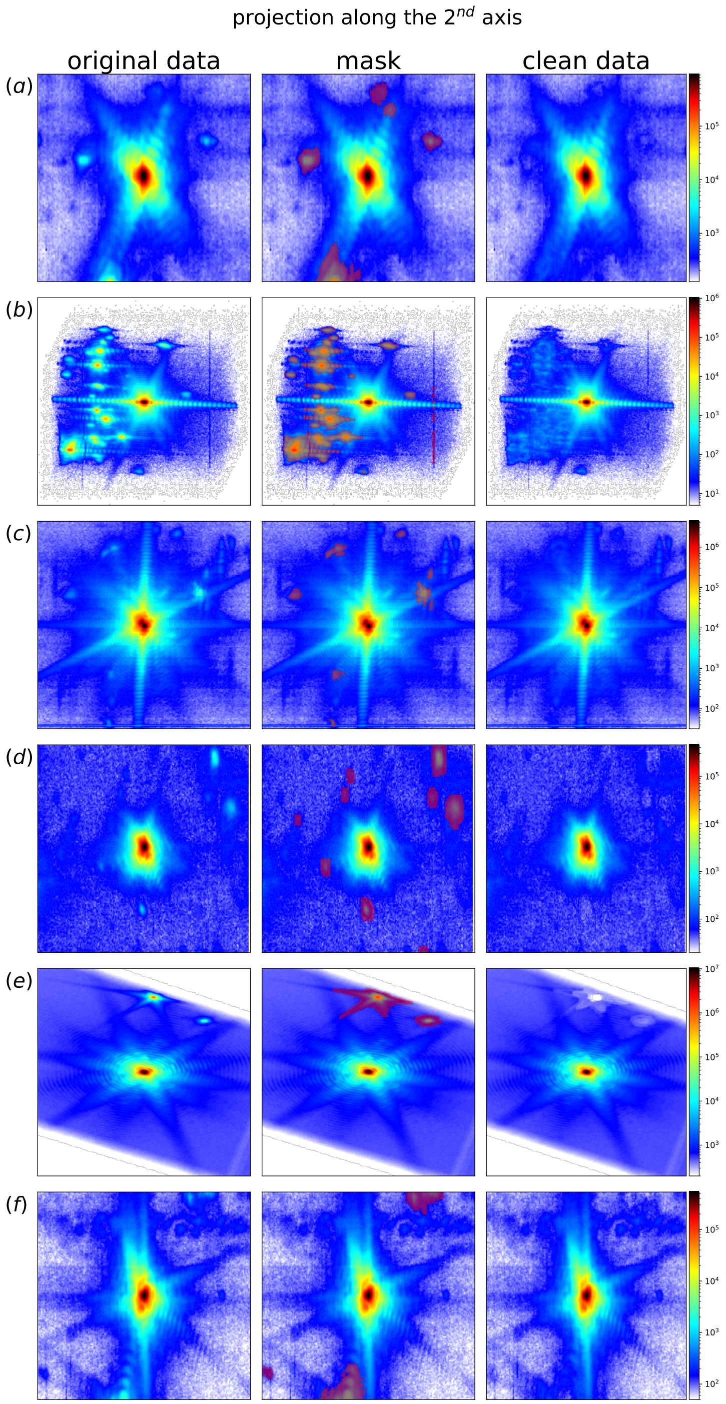

Supplementary Figure 6: From (*a*) to (*f*), different examples of BCDI data containing aliens. The 1st column shows the raw data, the 2nd column shows the alien mask and the 3rd column shows the cleaned data where masked pixels are put to 0.

Supplementary Section 6: Asymmetric peak masking

Our method works equally well on highly asymmetric peak, as shown for example in Supplementary Fig. **7** on a core-shell NiFe particle. Two aliens were artificially put by the authors in this BCDI array by adding cropped data coming from another particle. The alien positions were chosen randomly, leading to the 3D array projection shown in Supplementary Fig. **7**(*a*). Our clustering method was then applied to generate the mask shown in Supplementary Fig. **7**(*b*), demonstrating that the primary Bragg peak structure does not affect the masking process, provided the alien clusters remain disconnected from the central peak. The cleaned data is shown in Supplementary Fig. **7**(*c*), where the pixel values inside the mask were put to 0.

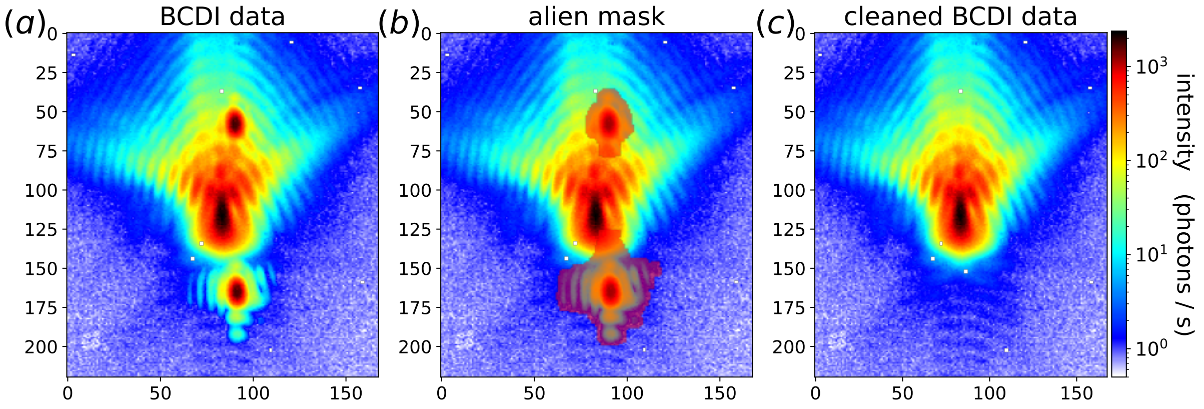

Supplementary Figure 7: (*a*) Highly asymmetric experimental data where 2 handmade aliens have been added at random positions. (*b*) Alien masking from our clustering method. (*c*) Cleaned data where the aliens pixel values were set to 0.

Supplementary Section 7: Pt sample

The sample used for the data shown in Fig. 4 of the main text is shown in Supplementary Fig. **8**. This sample is made of a patterned structure with Pt nanocrystals deposited on sapphire, consisting of periodic squares separated by 50μm (Supplementary Fig. **8**(*a*)). An isolated nanocrystal is located at the center of each square, separated from any other crystal by 25 μm. Despite this distance, the tails of our X-ray beam can still touch neighboring particles leading to the aliens observed in Fig. 4 of the main text.

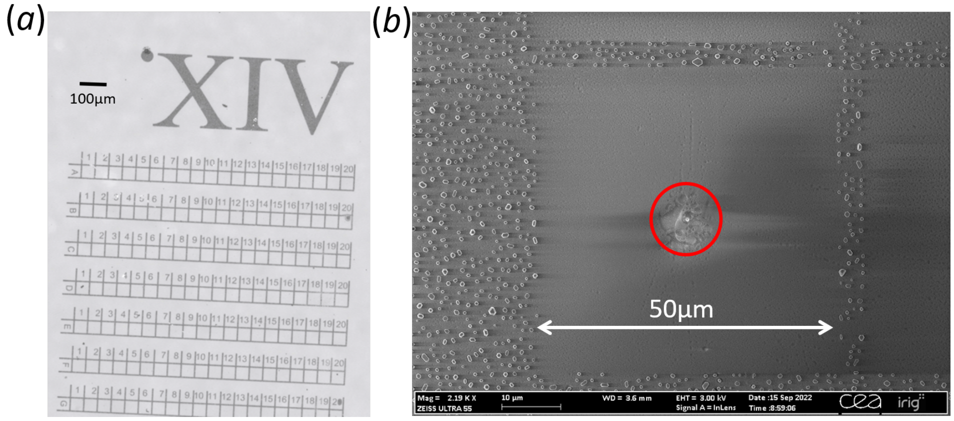

Supplementary Figure 8: (*a*) Pt nanocrystals on a sapphire substrate patterned using photolithography. (*b*) Zoom on one patterned square. The measured Pt nanocrystal is located at the center of the square (circled in red) and separated from other particles by 25 μm.

Supplementary Section 8: Diffuse scattering

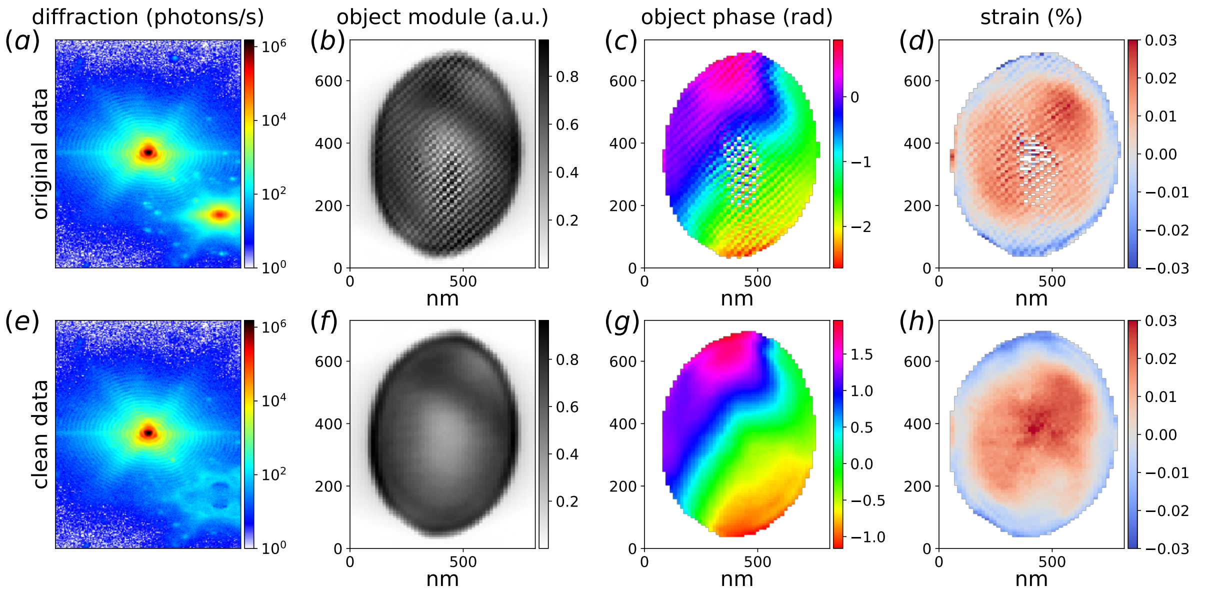

Supplementary Figure 9: Example of alien removal on a **111** Pt Bragg peak with a strong diffuse scattering. (*a*) to (*d*) correspond to the diffraction and reconstructed object without masking, while (*e*) to (*h*) correspond to the diffraction and reconstructed object after masking the alien diffuse peak.

Supplementary Figure **9** displays a detailed example of the alien masking on a diffraction pattern from a slightly misoriented Pt particle. The diffraction pattern contains an intense diffuse scattering peak coming from surrounding Pt particles (also shown in Fig. 4 of the main text). This diffuse peak causes significant oscillations in the object reconstruction, affecting both the module and phase (Supplementary Figs. **9**(*b*-*c*)). Therefore, the location of the surface of the particle is unclear and the calculated

strain (Supplementary Fig. **9**(*d*)) becomes unreliable. Furthermore, the surface of the object is slightly blurred by the presence of the aliens, which can be problematic, if one want to study surface reaction during in situ experiments. After masking the diffuse peak (Supplementary Fig. **9**(*e*)), the oscillations disappear in the reconstructed object (Supplementary Figs. **9**(*f*-*g*-*h*)).

Supplementary Section 9: Two aliens reconstruction

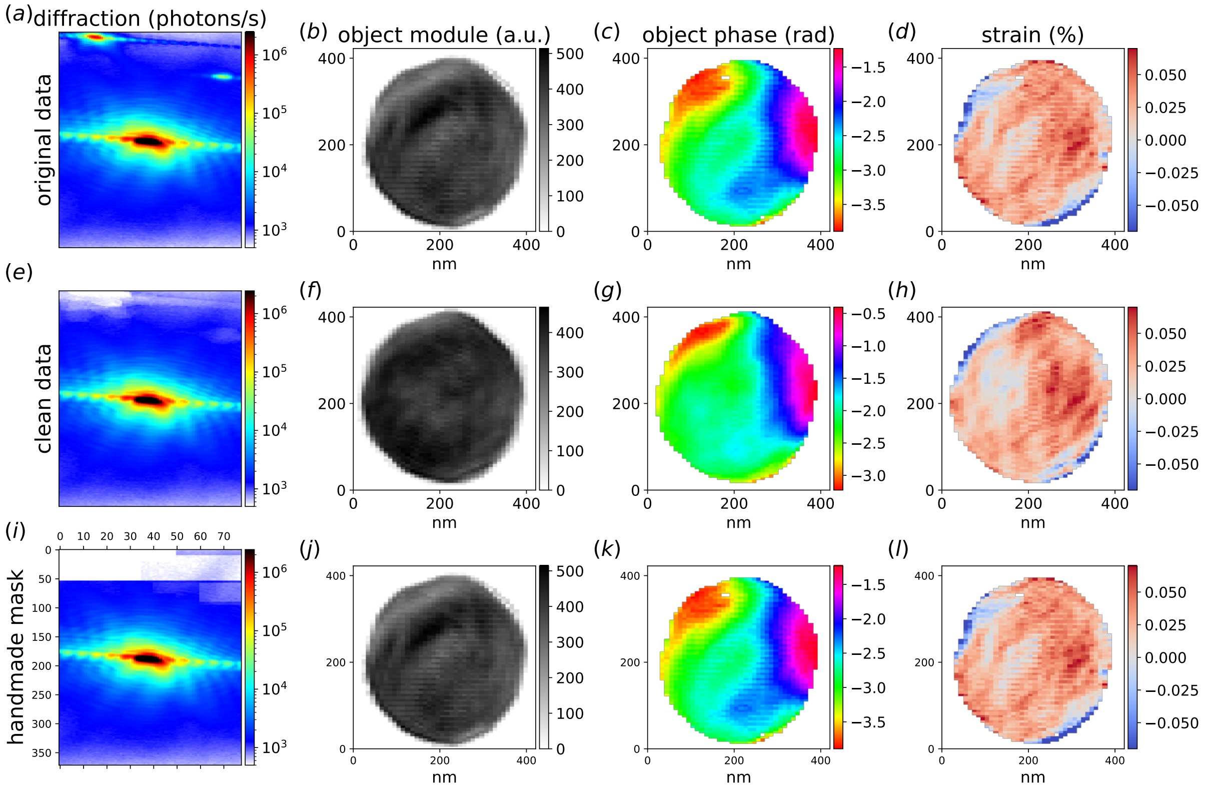

Supplementary Figure 10: Example of alien removal BCDI reconstruction on a particle with 2 aliens far from the Bragg peak center. (*a*) to (*d*) correspond to the diffraction and object reconstruction while (*e*) to (*h*) correspond to the diffraction and the reconstruction after masking the alien diffuse peak. (*i*) to (*l*) correspond to the diffraction and the reconstruction using a handmade rectangular mask.

Supplementary Figure **10** shows the reconstructions of a particle with 2 relatively strong aliens. The reconstruction of the object is still possible, but the aliens' presence induces short scale oscillations in the object phase and thus in the reconstructed out-of-plane strain component (Supplementary Figs. **10**(*c*-*d*)). As discussed above, the surface of the object (Supplementary Fig. **10**(*b*)) is slightly blurred by the presence of the aliens, which can be problematic.

Our masking method was used to remove the aliens resulting in the BCDI array shown in Supplementary Fig. **10**(*e*) with the corresponding reconstructed object (from (*f*) to (*h*)). The oscillation artefact

disappeared in the reconstructed phase and strain and the object surface is sharper.

For comparison, Supplementary Figs. **10**(*i*-*l*) show the diffraction pattern with a handmade mask made image-per-image using a combination of rectangular patches as well as its associated reconstructed object. However, the resulting mask is larger than the one created by our method, this leads to more empty space in the BCDI signal making the reconstruction more difficult.

Supplementary Section 10: Remaining alien signal

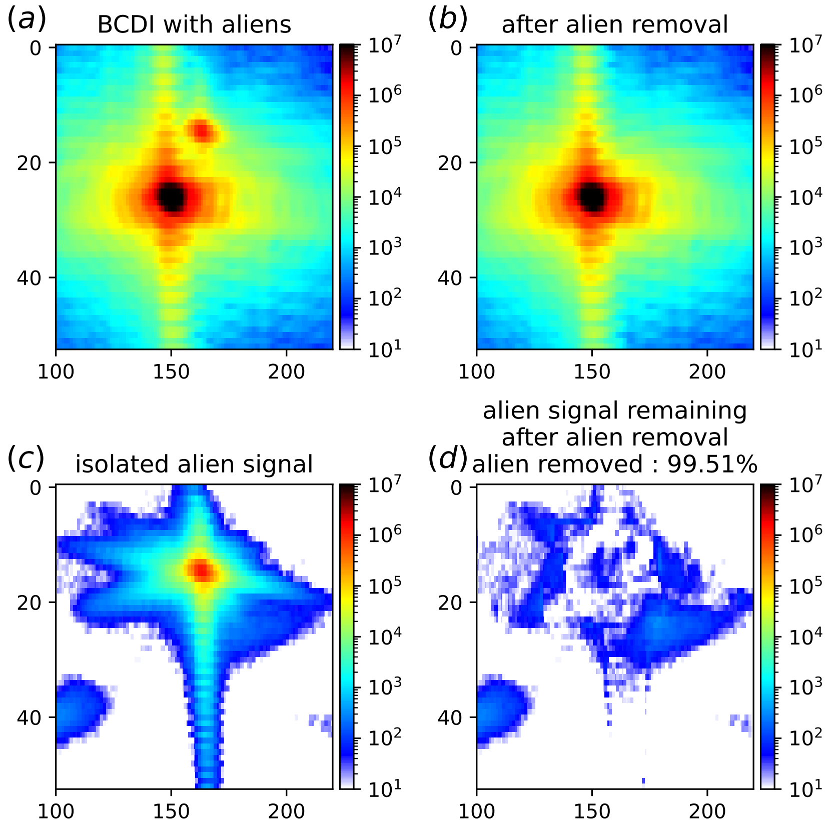

Supplementary Figure 11: (*a*) BCDI data with an alien signal (in log scale). (*b*) Same data after alien removal using our method. (*c*) Same alien signal isolated by cropping the BCDI array. (*d*) Remaining alien signal after removal with our method. 99.51% of the alien intensity is removed.

One can observe in the Figure 4 of the main text that our algorithm does not remove all the alien parasitic signal. However, the remaining alien signal is very low after cleaning, since the brightest part (central part of the alien peaks) is removed. We emphasize that in Figure 4, the BCDI intensity is shown in log-scale. In order to assess the ratio of alien signal remaining after data processing using our method, we considered the experimental BCDI data shown in Supplementary Fig. **11**(*a*). This data

contains our main central Bragg peak as well as a small alien signal on the top right. Supplementary Fig. **11**(*b*) shows the same array after alien removal with our method. In (*c*), we have isolated the alien signal, cropped the array along the rocking curve direction in order to catch only the alien signal. In (*d*), we show the same isolated alien signal after alien removal. While some part of the alien still remains, 99.51% of the alien intensity is removed by our clustering technique.

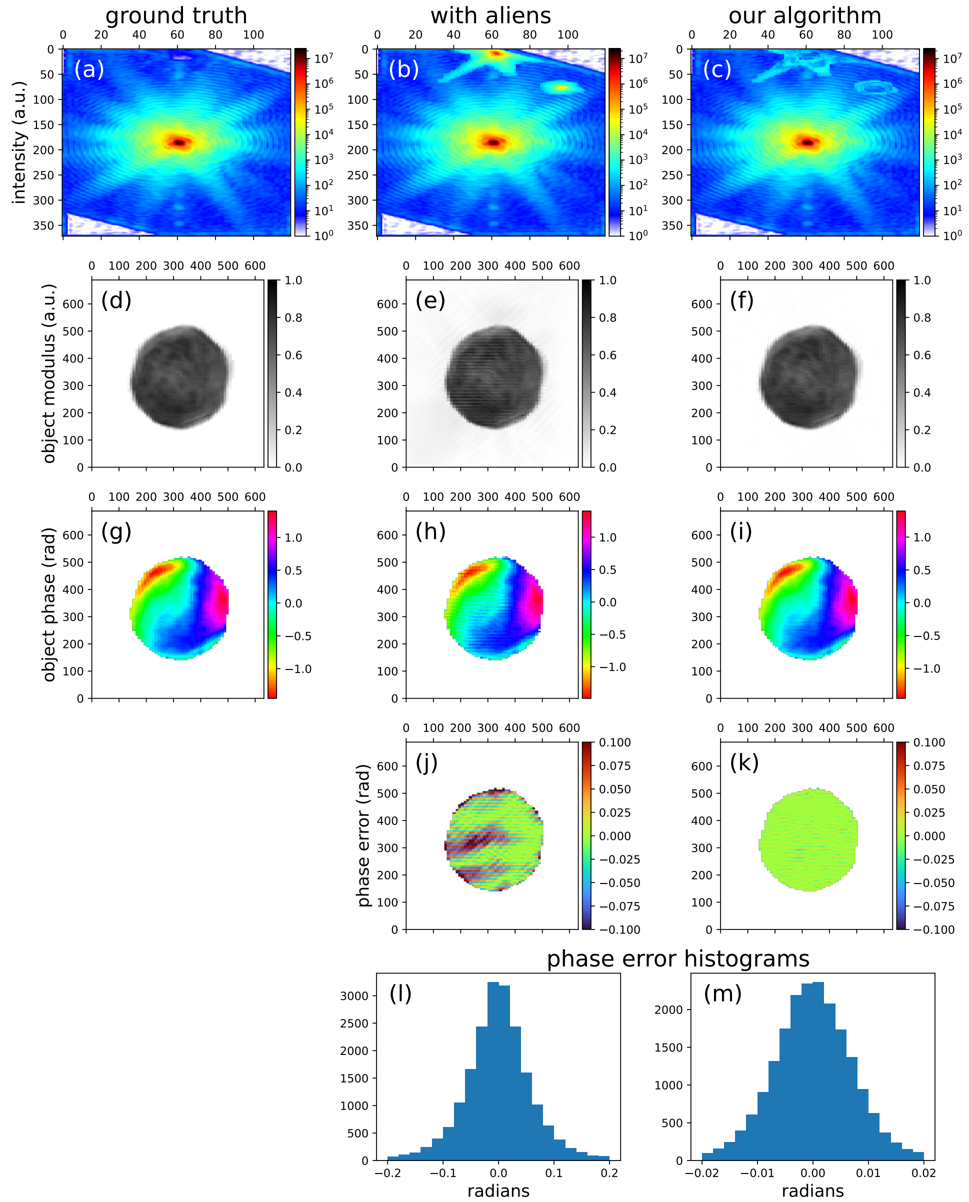

Supplementary Figure 12: (*a*) experimental BCDI data (*b*) on which simulated alien signal is added (*c*) and then removed partially using our method. (*d*-*i*) corresponding reconstructed objects. (*j*) phase error induced by the alien and (*k*) after using our cleaning method. (*l*-*m*) Corresponding phase error histograms.

We show in Supplementary Fig. **12** the effect of the remaining alien signal on the reconstructed

phase. We used experimental BCDI data (*a*) on which we added a simulated alien signal (*b*) that we partially removed using our method (*c*). The corresponding objects are shown in (*d*-*i*) showing small oscillation artefacts in the presence of aliens. The phase errors are shown in (*j*-*k*) along with the phase error histograms (*l*-*m*). Despite weak intensity regions of the alien signal remaining after using cleaning method, this has very little effect on the reconstructed phase with a phase error ± 0.02 radians (*m*) which is negligible compared to the ground truth phase variations ± 1 radians (*g*).

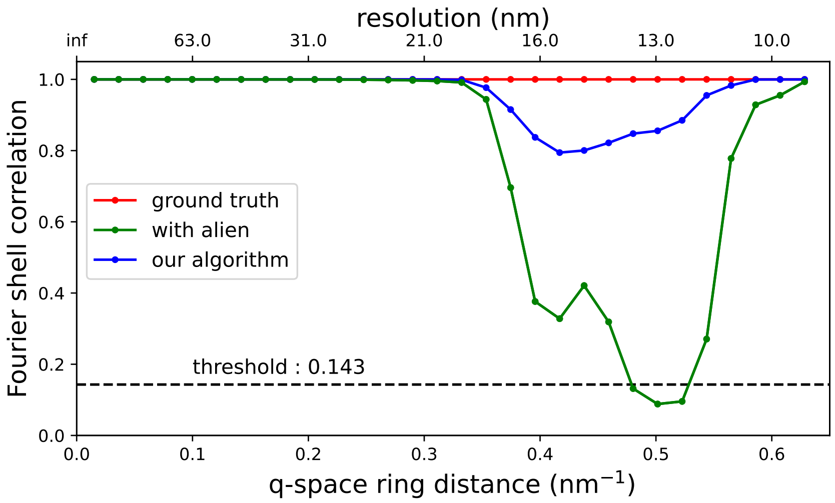

Fourier shell correlation analysis for the simulated data shown in Supplementary Fig. **12**

In order to evaluate the spatial resolution on the simulated data shown in Supplementary Fig. **12**, we perform a Fourier Shell Correlation (FSC) analysis as illustrated in the figure above. As expected, the presence of aliens decreases the object resolution down to 14nm (in green). Despite the fact that our algorithm doesn't remove all alien signal, it is still able to remove enough of it to mitigate the aliens' artifacts and keep a FSC coefficient well above the 0.143 threshold.

Supplementary Section 11: Spatial resolution

In order to assess the spatial resolution of the reconstructed object after alien removal using our method shown in Figure 4(*e*-*f*), we calculated the Fourier shell correlation on two different reconstructions. The result is shown in Supplementary Fig. **13**, which gives a spatial resolution of 5.8 nm .

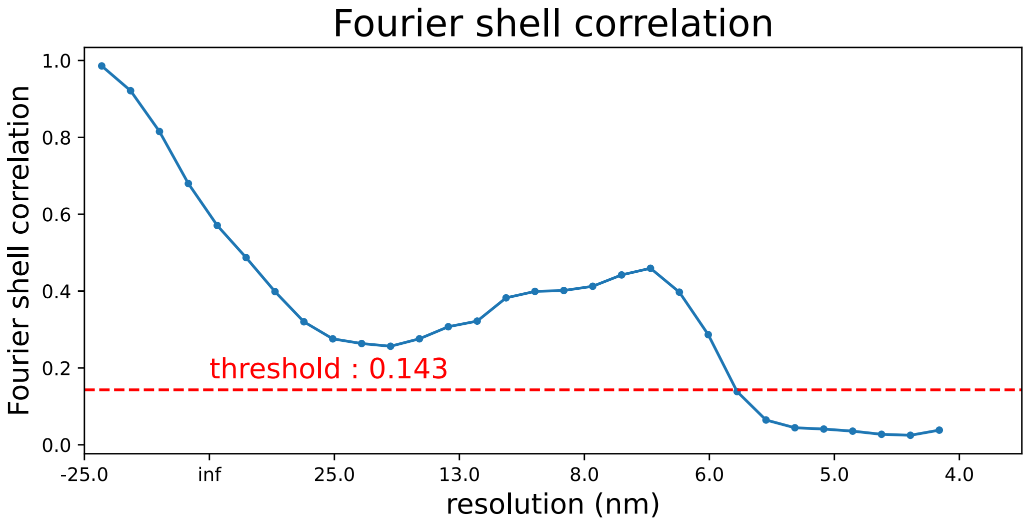

Supplementary Figure 13: Fourier shell correlation for the object reconstruction after alien removal shown in the Main text Figure 4(*e*-*f*).

Supplementary Section 12: Comparison with auto_alien1

In this section, we compare the auto_alien1 function used in Pelzer et al. (2021) and available at *https://github.com/AdvancedPhotonSource/cohere.git* with our method. In Supplementary Fig. **14**, we show the results of auto_alien1 on different experimental BCDI data using the default set of parameters. Although the same parameters can be used to clean up most data, in some difficult cases some alien signals still remain. In Supplementary Figs. **14**(*d*-*e*), the aliens are close to the central Bragg peak and the background is noisy, in that case auto_alien1 with default parameters is not able to catch all parasitic signals. Supplementary Fig. **14**(*g*) contains high-resolution BCDI data with numerous aliens (see Supplementary Fig. **6**(*b*)). Although most of them have been removed, a few remain.

Supplementary Figure **15** shows a grid search of auto_alien1 results for different values of the *amp_threshold parameter*. As observed, increasing *amp_threshold* can help removing aliens. However, this also removes diffracted intensity far from the Bragg peak center, thus reducing the reconstructed object spatial resolution.

Supplementary Figure **16** shows a second grid search on the *size_threshold* parameter. Despite not removing all aliens for difficult BCDI data (Bragg 3 and 4), increasing size_threshold up to 0.5 leads to the best results.

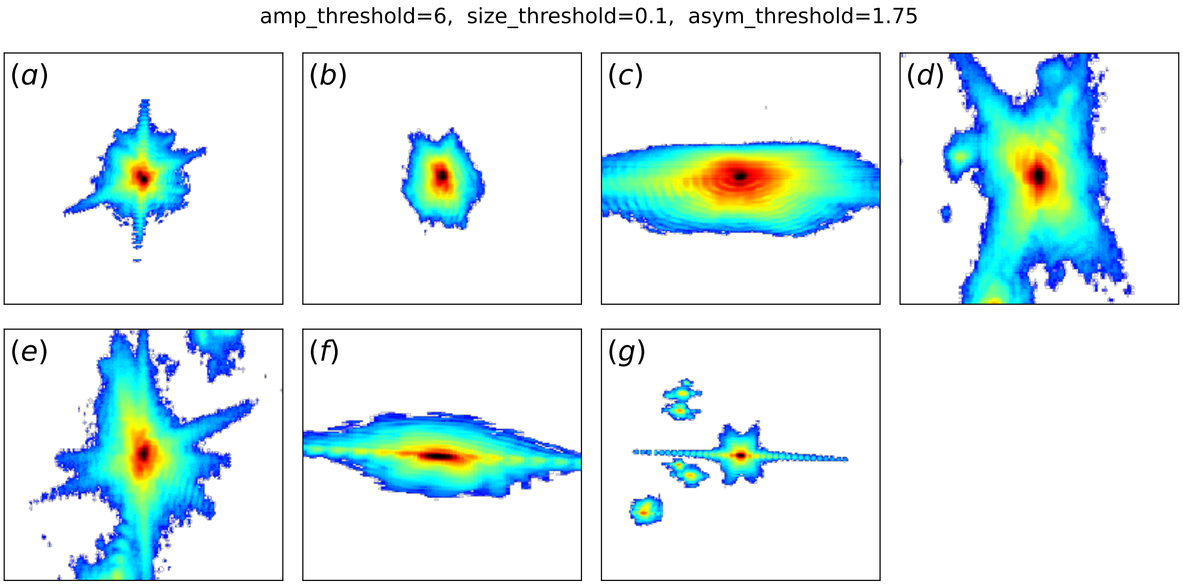

Supplementary Figure 14: auto_alien1 using default parameters amp_threshold, size_threshold and asym_threshold on several experimental data containing aliens (shown in Supplementary Fig. **6**). The same set of parameters does not always work for each BCDI data (panels (*d*), (*e*) and (*g*)).

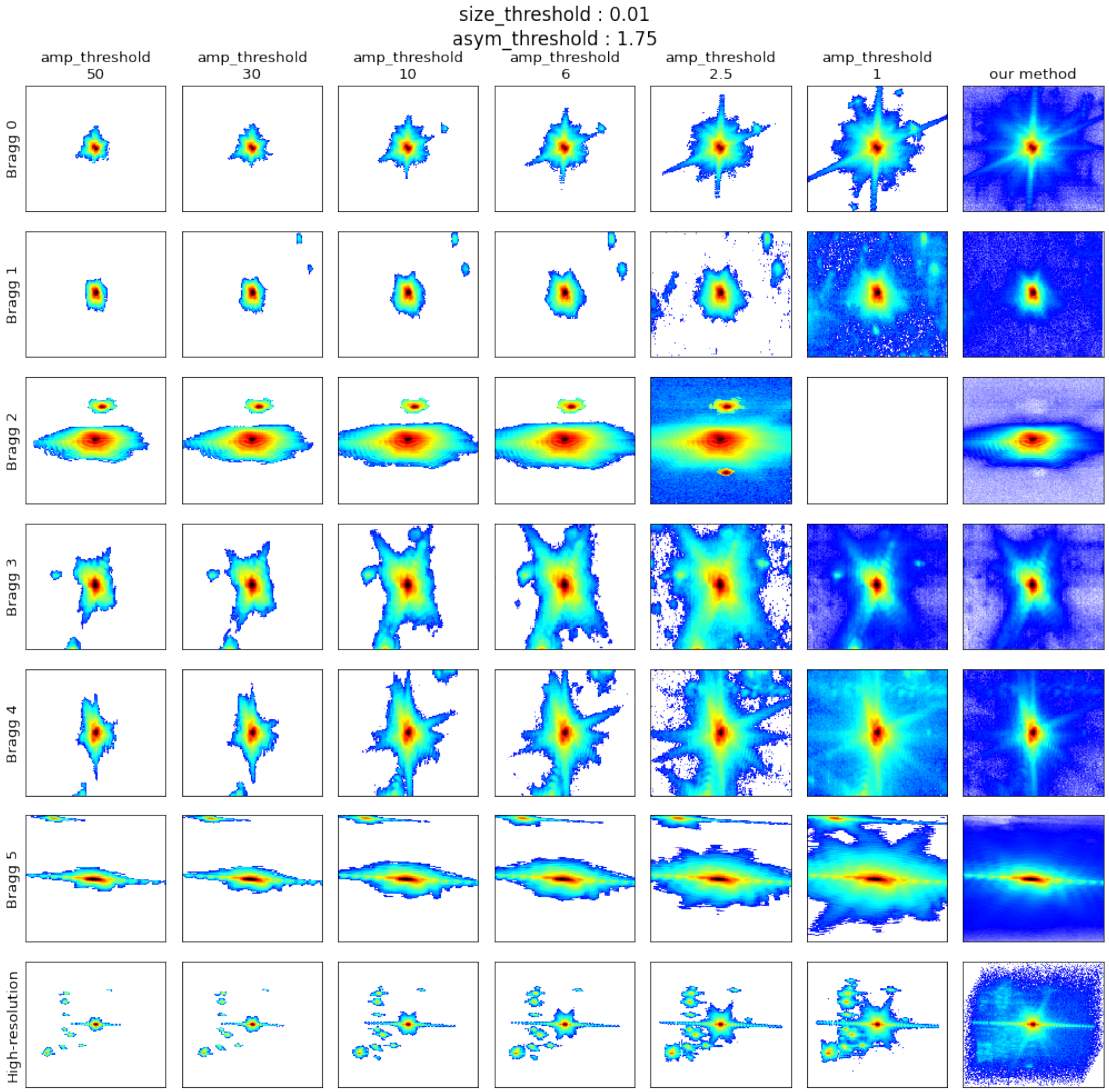

Supplementary Figure 15: Grid search of the optimal amp_threshold parameter in auto_alien1 applied on different experimental BCDI data. We can observe that the same set of parameters does not always remove all alien signals for each BCDI data.

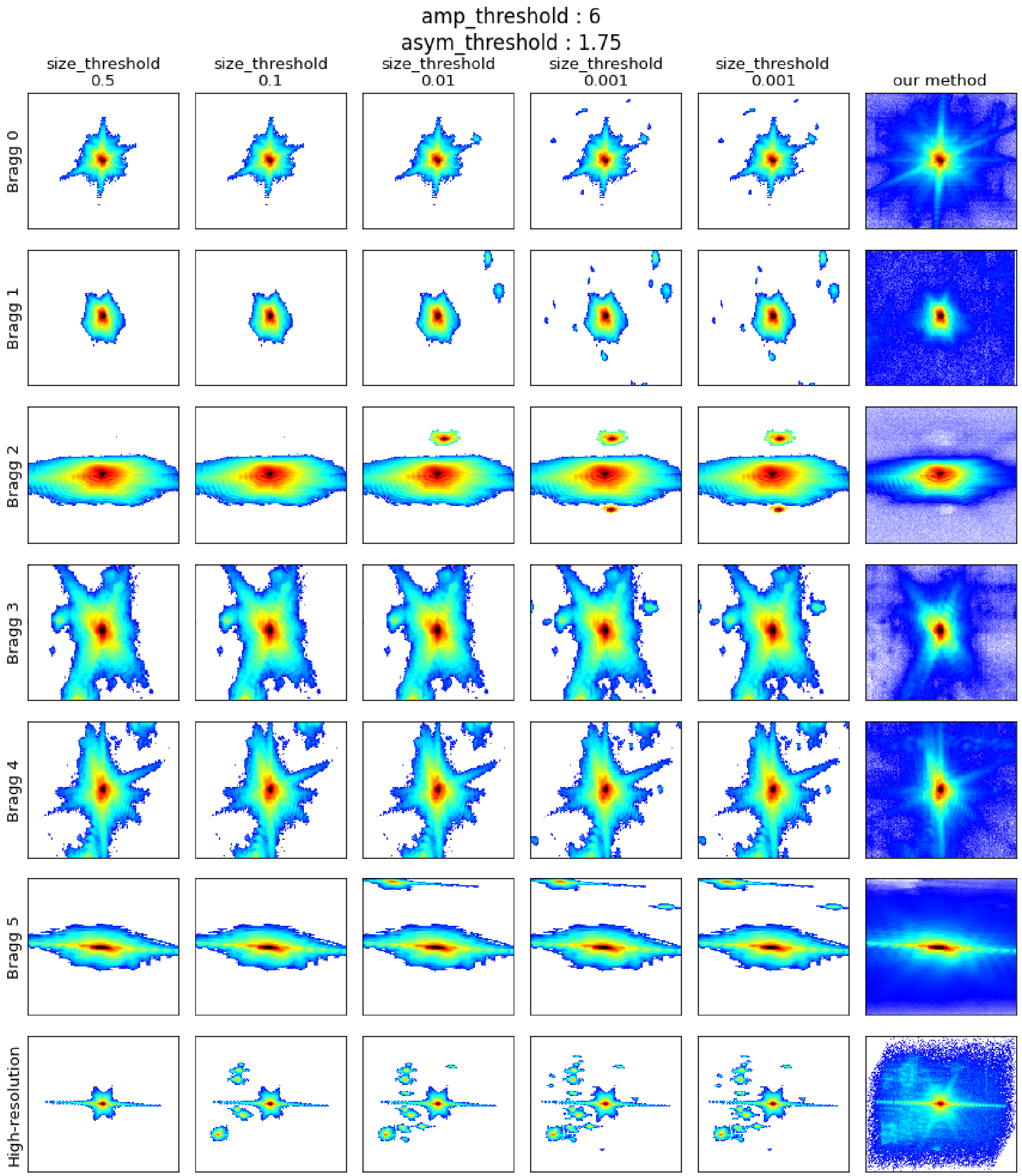

Supplementary Figure 16: Grid search of the optimal size_threshold parameter for auto_alien1. The largest size_threshold value (0.5) gives the best results. However, for some noisy data (Bragg 3 and Bragg 4), increasing size_threshold does not remove all the signals from the aliens.

Supplementary Section 13: Simulated object

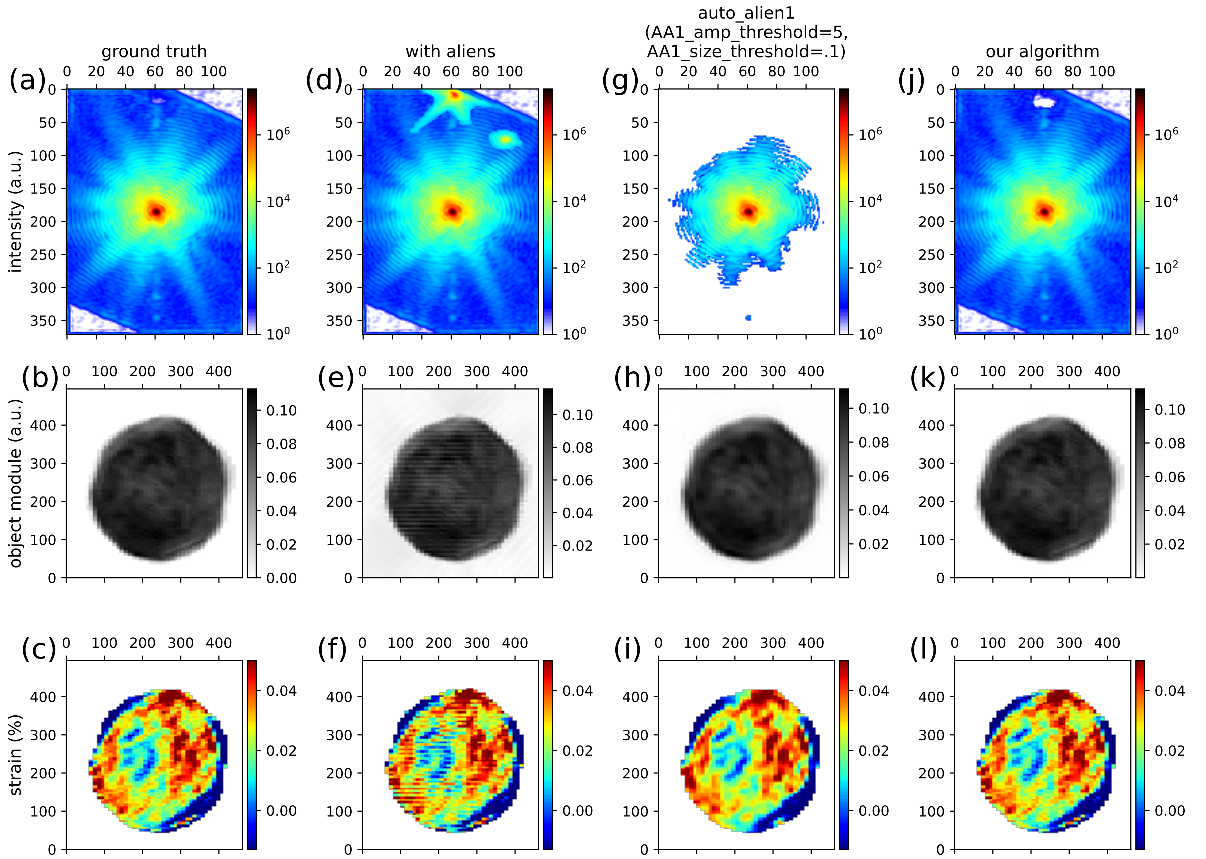

Supplementary Figure 17: (*a*)-(*b*)-(*c*) Simulated diffraction pattern of a clean object with its reconstruction (module and strain). (*d*)-(*e*)-(*f*) Same object with additional alien signals. Some oscillations artefacts are visible in the strain. (*g*)-(*h*)-(*i*) Alien removal using auto_alien1 where high-q information is lost. (*j*)-(*k*)-(*l*) Alien removal using our method.

In order to compare auto_alien1 with our method on the real-space object, we used the simulated object shown in Supplementary Fig. **17**(*a-b-c*). This object corresponds to the reconstruction of experimental data in order to be as close as possible to what is typically measured in BCDI. We then added two alien signals as shown in Supplementary Fig. **17**(*d*) leading to high-frequency oscillation artefacts on the object's strain map in Supplementary Fig. **17**(*f*). Supplementary Fig. **17**(*g*) shows the result of using auto_alien1 to remove the alien signal. However, auto_alien1 also removes high-q diffracted intensity leading to a loss in real-space resolution. Finally, Supplementary Fig. **17**(*j*) shows the result of alien removal using our method.

Supplementary Fig. **18**(*a*) shows a zoom on a small region of the strain map. Panel (*b*) show the same region after adding alien signals leading to oscillation artifacts. Panels (*c*)-(*d*) illustrate the effect of alien removal using auto_alien1 and our method. While auto_alien1 "smooths" the strain map by removing high-q diffracted intensity, our method is able to both remove alien signals rapidly and recover a strain map with the same spatial resolution as the ground truth.

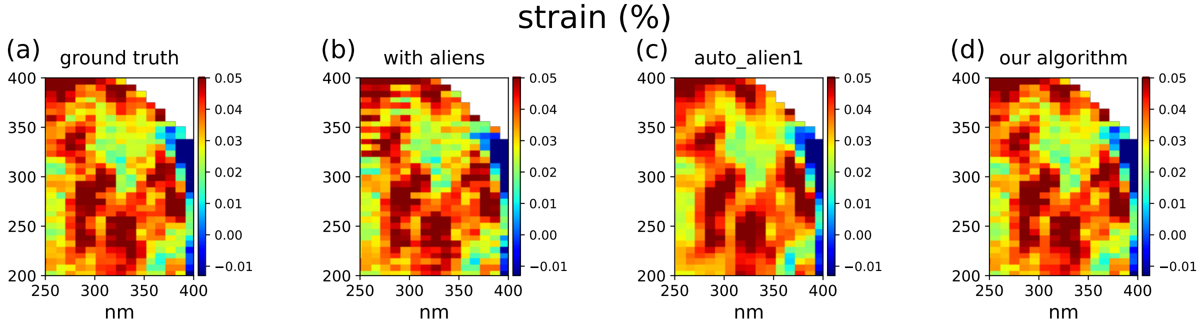

Supplementary Figure 18: (*a*) Zoom on the strain map of the simulated object. (*b*) Strain map after adding alien signal. (*c*) Strain map after alien cleaning using auto_alien1. One can observe a decrease of the spatial resolution. (*d*) Strain map after alien removal using our method.

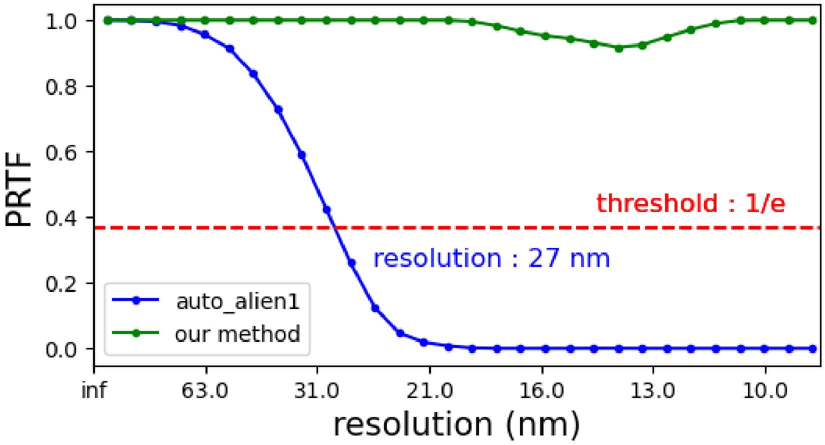

Supplementary Figure 19: Phase Retrieval Transfer Function (PRTF) for the real-space object after alien removal using auto_alien1 (in blue) and our method (in green).

Supplementary Figure **19** shows the object's resolution calculated using the Phase Retrieval Transfer Function (PRTF) after cleaning the alien signals using auto_alien1 (blue curve) and our method (green). Since high-q signals are removed using auto_alien1, the real-space resolution decreases to 27 nm. However, using our method, high-q signal is conserved and, according to the PRTF, the reconstructed object resolution is limited by the voxel size (10 nm). This is probably due to the fact that we still observe diffracted intensity up to the borders of the BCDI array in Supplementary Fig. **17**(*j*).

Supplementary Section 14: Mask threshold overlap

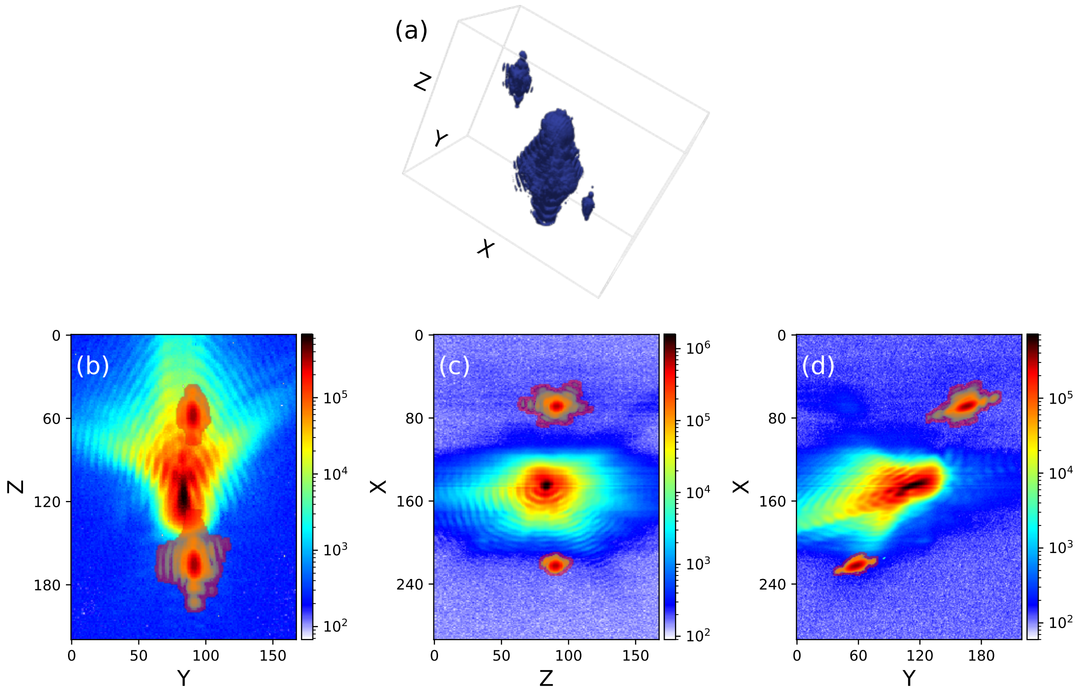

Supplementary Figure 20: (*a*) 3D iso-surface representation of high-strain BCDI data. A central peak and 2 alien signals are visible. (*b*)-(*d*) 2D projections of the 3D BCDI array along each axis with the corresponding alien mask using our method shown in semi-transparent red.

Representing the 3D BCDI data as 2D projections can lead to a wrong impression of overlap between our created alien mask and the central peak, such as in Supplementary Fig. **6**(*a*)-(*c*)-(*f*) and Supplementary Fig. **7**(*b*). However, this impression is only due to the 2D projection representation and no overlap occurs on the 3D array. To illustrate this, we show in Supplementary Fig. **20**(*a*) high-strain BCDI data from Supplementary Section 6 as a 3D iso-surface. We can observe that the 2 alien signals are clusters far from the central peak. In (*b*)-(*c*)-(*d*), we show the data projection along each axis with the corresponding alien mask in semi-transparent red. We can observe that our alien mask doesn't have an overlap with the central peak in 3D.

Despite the occasional problem of confusing an overlap in the 2D projections, we chose this display for cluster and mask threshold selection since it's much faster than a 3D representation (especially for large BCDI arrays).

Supplementary Section 15: Reason for the occurrence of alien signal

Sample preparation such as patterning using photolithography (Supplementary Figure **8**) in order to isolate a single nano-particle can help reduce the probability to measure an alien signal. However, this is not always sufficient and we can still observe parasitic signals for several reasons.

In typical BCDI experiments, the X-ray beam is focused via a KB mirror, a Fresnel zone plate or a set of Beryllium (Be) lenses. Despite the small beam size at focal ($\sim 1\mu$m), the beam tails can be large and still have enough intensity to catch other particles inducing aliens in our measurements. In Supplementary Figure **21**, we show a ptychography reconstruction of a focused X-ray beam by a set of Be lenses. The focused beam size is around 350μm. However, as shown in the zoom inset, the beam tails are still relatively large and with enough intensity to catch other neighbor particles. In typical BCDI experiment, the beam size is often up to 1μm and the beam tails can catch particles that are more than 10μm away from the center.

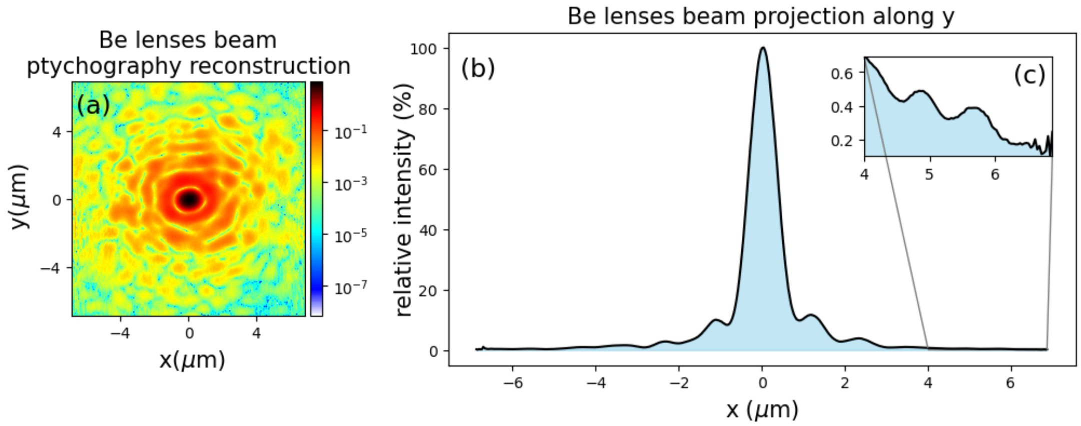

Supplementary Figure 21: (a) Ptychography reconstruction of the X-ray beam focused by a set of Beryllium lenses (in log scale). (b) Projected sum of the beam along y. Beam FWHM is 350μm however the beam tails intensity is not negligible as shown in the inset (c)

Furthermore, due to the beam projection on the sample, the illuminated surface is larger than the beam size along the beam direction. For example, in the case of the Pt 111 peak measured at 12keV, the Bragg angle is only 13.2 degrees, thus a projection factor of $1/sin(13.2°) \approx 4.4$. A typical 1μm beam size is increased up to 4.4μm (the beam tails going even further) and can illuminate several neighbor particles leading to the observed alien peaks as shown in Figure 4 of the main text.

Finally, sample preparation using photolithography is not always a solution and can be time-consuming. Often, the nano-particles orientation is random and it becomes more efficient to perform

BCDI on a dense powder leading to many alien signals. Using our cleaning method, one can perform fast ex-situ measurements to characterize a sample without the need for any time-consuming sample preparation.
